# Assessing the impact of gestational age of donors on the efficacy of amniotic epithelial cell-derived extracellular vesicles in experimental bronchopulmonary dysplasia

**DOI:** 10.1186/s13287-022-02874-4

**Published:** 2022-05-12

**Authors:** Dandan Zhu, Mirja Krause, Tamara Yawno, Gina D. Kusuma, Renate Schwab, Mehri Barabadi, Amina S. Maleken, Siow T. Chan, Rod Hunt, David Greening, Euan M. Wallace, Rebecca Lim

**Affiliations:** 1 The Ritchie Centre, Hudson Institute of Medical Research, Clayton, VIC 3168 Australia; 2 Department of Obstetrics and Gynaecology, Monash University, Clayton, VIC 3168 Australia; 3 Department of Paediatrics, Monash University, Clayton, VIC 3168 Australia; 4 Baker Heart and Diabetes Institute, Melbourne, VIC Australia; 5 Department of Biochemistry and Genetics, La Trobe Institute for Molecular Science, La Trobe University, Bundoora, VIC Australia; 6 Central Clinical School, Monash University, Clayton, VIC Australia; 7 Baker Department of Cardiometabolic Health, University of Melbourne, Parkville, VIC Australia

**Keywords:** Human amniotic epithelial cells, Extracellular vesicles, Prematurity, Bronchopulmonary dysplasia

## Abstract

**Background and rationale:**

Extracellular vesicles (EVs) are a potential cell-free regenerative medicine. Human amniotic epithelial cells (hAECs) are a viable source of cell therapy for diseases like bronchopulmonary dysplasia (BPD). However, little is known about the impact of gestational age of the donor on the quality of hAEC-derived EVs.

**Aims:**

To determine the impact of gestational age on hAEC-derived EVs in experimental BPD.

**Results:**

Term hAEC-derived EVs displayed a significantly higher density of surface epitopes (CD142 and CD133) and induced greater macrophage phagocytosis compared to preterm hAEC-EVs. However, T cell proliferation was more significantly suppressed by preterm hAEC-EVs. Using a model of experimental BPD, we observed that term but not preterm hAEC-EVs improved tissue-to-airspace ratio and septal crest density. While both term and preterm hAEC-EVs reduced the levels of inflammatory cytokines on postnatal day 7, the improvement in lung injury was associated with increased type II alveolar cells which was only observed in term hAEC-EV treatment group. Furthermore, only neonatal term hAEC-EVs reduced airway hyper-responsiveness, mitigated pulmonary hypertension and protected against right ventricular hypertrophy at 6 weeks of age.

**Conclusion:**

Term hAEC-EVs, but not preterm hAEC-EVs, have therapeutic efficacy in a mouse model of BPD-like lung injury. Therefore, the impact of donor criteria should be considered when applying perinatal cells-derived EV therapy for clinical use.

## Background

Extracellular vesicles (EVs) derived from pro-regenerative cells are being considered as an alternative for regenerative medicine, serving as non-living carriers of therapeutic proteins, lipids, mRNA and microRNA [1]. At the time of this manuscript preparation, 31 clinical trials have been registered with the National Institute of Health (NIH), investigating EV-based therapeutics (https://clinicaltrials.gov). These include five trials (NCT02138331, NCT03437759, NCT04213248, NCT04798716 and NCT04134676) using EVs isolated from umbilical cord blood or umbilical cord-derived mesenchymal stromal/stem cells (UC-MSCs).

Perinatal stem/stem-like cells derived from gestational tissues include a few regenerative cell types such as hematopoietic stem/progenitor cells (HSCs/HPCs), endothelial progenitor cells (EPCs), mesenchymal stem/stromal cells (MSCs) and human amniotic epithelial cells (hAECs) [2]. Recent studies have shown that MSCs and hAECs are promising cell therapies for bronchopulmonary dysplasia (BPD) [3]. There are now 12 NIH-registered clinical trials using UC-MSCs as a treatment for BPD (https://clinicaltrials.gov).

The therapeutic effects of perinatal stem cells from preterm birth remain a subject of debate. UC-MSCs from preterm birth were shown to have beneficial effects in treating lung injury [4]. However, others have found that even when term and preterm cord blood cells reduced preterm white matter injury, the mechanisms of neuroprotection appear different following term vs. preterm cord blood administration [5]. There has been limited information to date on how gestational age influences EV quality and potency. EVs derived from UC-MSCs have been shown to have therapeutic effects in experimental models of BPD. Kourembanas et al. discovered that MSC-EVs derived from term umbilical cord Wharton’s jelly improved lung function and ameliorated secondary pulmonary hypertension [6]. Bhandari et al. found that MSC-EVs derived from early gestational age umbilical cord also improved lung structure and reduced inflammation in a mouse model of BPD [4]. Lim et al. suggested that there was an inverse correlation between the MSC-EV production and developmental maturity of the donor tissues; however, this did not affect the efficacy [7]. It is therefore important to understand if and how the gestational age of the donor influences the yield and potency of EVs derived from perinatal stem/stem-like cells.

The anti-inflammatory and anti-fibrotic effects of term hAECs in lung repair have been previously shown in several mouse and sheep lung injury models [8–10]. hAECs play several important roles in maternal–foetal tolerance during pregnancy, inducing expansion of regulatory T cells and limiting chronic inflammatory diseases [11], polarize macrophages from classical M1 to alternative M2 phenotypes, and encourage phagocytic activity [12]. Recently, hAECs have been shown to have potent immunomodulatory properties associated with their constitutive production of ectoenzymes that are able to drive expansion of regulatory B cells and suppress NK cell activity [13]. hAECs from term, healthy pregnancies appear to be a potent source of cell therapy for BPD [14], which is a major respiratory complication associated with extreme prematurity. Most recently, hAECs have progressed into clinical trials for BPD [15–17].

Little is known about the impact of gestational age of the donor on EVs derived from hAECs (hAEC-EVs). To address this gap in research, we used a combination of in vitro potency assays and murine model of experimental BPD [18] to: (1) characterize term and preterm hAEC-EVs; and (2) compare the therapeutic potential of term hAECs, term hAEC-EVs and preterm hAEC-EVs in experimental BPD.

## Methods

### hAEC isolation

hAECs were isolated from amnions collected after caesarean sections, and cell purity was determined by flow cytometry as previously described [19]. Cell viability was determined using Trypan blue exclusion, where cells of > 80% viability were used in this study. Preterm birth was defined as birth before 37 completed weeks of gestation; term was birth after 37 completed weeks of gestation. The mean gestational age of preterm donors was (Mean ± SEM) 32.6 ± 1.4 weeks and 38.4 ± 0.6 weeks for term donors. All isolated cell lines were de-identified in accordance with guidelines on human research ethics as set out by the National Health and Medical Research Council.

### hAEC-EV isolation

Ten million hAECs were plated into a T175 flask with 25 mL UltraCULTURE™ + 1%L-glutamine serum-free media (UltraCULTURE™; LONZA, USA). Cells were cultured for 4 days at 37 °C, 95% humidified air and 5% CO_2_, and then the conditioned media were collected for EV isolation using serial ultracentrifugation as previously described [20].

### hAEC-EV characterization

The characterization of hAEC-EVs (*n* = 5) was performed in accordance with the Minimal Information for Studies of Extracellular Vesicles (MISEV) guidelines [21]. The morphology of hAEC-EVs was determined by transmission electron microscopy (TEM). Protein concentration was determined using a bicinchoninic acid (BCA) assay (Pierce BCA Protein Assay Kit, Thermo Fisher Scientific, USA). Nanoparticle tracking analysis (NTA, NanoSight, UK) was used to measure particle numbers and size distribution. Surface epitopes were determined using the MACSPlex Exosome Kit (130-108-813, Miltenyi Biotec, USA). Samples were processed according to each manufacturer’s instructions. Proteome composition of hAEC-EVs was determined using proteomic analysis as previously described [22].

### T Cell proliferation assay

Freshly isolated CD4^+^ human T cells were labelled with CellTrace Violet (CTV, Thermo Fisher Scientific, USA), activated with Dynabeads (Thermo Fisher Scientific, USA) coated with human anti-CD3/CD28 (beads/cell = 1:1), and cultured with/without hAEC-EVs for 96 h before analysed with fluorescence-activated single-cell sorting (FACS analysis).

### Macrophage phagocytosis assay

Immortalized monocyte-like cells, THP-1, were stimulated with Phorbol 12-myristate 13-acetate (PMA, 200 nM/mL) for 72 h before hAEC-EVs were added and incubated for another 24 h. pHrodo Red *E. coli* BioParticles (100ug/mL, Thermo Fisher Scientific, USA) was added and incubated for 2 h, and then the fluorescent intensity was measured at 560/585 nm.

### Animals and experimental groups

**Fig. 1:**
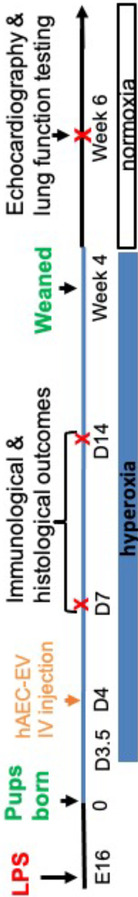
Flow chart of experimental induced BPD bronchopulmonary dysplasia (BPD) model. C57/BL6 mice were time-mated and on day 16 of pregnancy (E16) injected with either 0.1 μg lipopolysaccharide (LPS) in 5 μL saline or an equal volume of vehicle into each amniotic sac. Pups were then allowed to deliver naturally at term. The pups were placed either in normoxia or in hyperoxia chambers on PND3.5 (12 h before hAEC treatment). On PND4, either hAECs (human amnion epithelial cells, 100,000 cells) or EVs (term/preterm extracellular vesicles, 10 μg) or saline was injected intravenously through the superficial temporal vein using the automated microinjection system as per the intra-amniotic injections. One cohort of mouse pups were culled on either PND7 or PND14. Another cohort of mouse pups were kept in hyperoxia chamber until PND28 when they were weaned and transferred to standard housing facilities. Animals were culled at 6 weeks of age

C57/BL6 mice were time-mated and used for these experiments. Mice were kept in 12-h light/dark cycle and had free access to water and food. On day 16 of pregnancy (embryonic; E16), mice were injected with either 0.1 μg lipopolysaccharide (LPS; Sigma, St. Louis, MO) in 5 μL saline or an equal volume of vehicle into each amniotic sac. Pups were then allowed to deliver naturally at term (E21). Newborn pups were exposed to either normoxia (fraction of inspired oxygen; FiO2 = 0.2) or hyperoxia (FiO2 = 0.65) from postnatal day 3.5 (PND3.5). On PND4, pups received either term hAECs (the previously shown effective therapy [23]), term hAEC-EVs, preterm hAEC-EVs, or saline via intravenous delivery through the superficial temporal vein. For short-term studies, mouse pups were culled on PND 7 and 14. For long-term studies, pups were kept in hyperoxia chamber until PND28 when they were weaned and transferred to standard housing facilities, before experiments were terminated at 6 weeks of age. To prevent maternal oxygen toxicity, nursing dams were rotated between chambers every 2 days. A representation of the experimental design is shown in Fig. 1. Term and preterm hAEC-EVs used in animal work were pooled from five cell lines each. In total, 50 dams were used, and the survival rate of the offspring was 75% due to the cannibalism of the C57Bl6 dams and neonates.

Experimental groups:

- Intra-amniotic saline + normoxia + intravenous saline,
- Intra-amniotic LPS + hyperoxia + intravenous saline,
- Intra-amniotic LPS + hyperoxia + intravenous term hAECs (100,000 cells),
- Intra-amniotic LPS + hyperoxia + intravenous term hAEC-EVs (10 μg),
- Intra-amniotic LPS + hyperoxia + intravenous preterm hAEC-EVs (10 μg).

### Lung histology and immunohistochemistry staining

Mouse pup lungs were inflated and fixed with 4% (w/v) paraformaldehyde. Paraffin sections were cut at 5 μm thickness and stained with haematoxylin and eosin (H&E). Eight to ten non-overlapping sequential fields of view were taken at 200 × magnification. Lung development and alveolar simplification were determined by measuring tissue-to-airspace ratio as previously described [24]. Hart’s staining and immunohistochemistry, von Willebrand factor (vWF), alpha-smooth muscle actin (α-SMA), bronchioalveolar stem cell (BASC) and type II alveolar cells (AT2s) were performed, and image analysis was completed as previously described [23–25]. The ratio of secondary septal crests to tissue was calculated, pulmonary vessel numbers were determined by counting the number of vWF-positive vessels stratified by diameter (≤ 50 and > 50 µm), and the average fluorescence intensity of α-SMA by perimeter for each pulmonary artery was calculated using ImageJ (NIH, Bethesda, MD). The average number of BASCs per terminal bronchiole was determined by manual counting, while the percentage number of AT2s was determined using Imaris software (Bitplane, Zurich, Switzerland).

### Lung lysate levels of inflammatory cytokines

Assessment of inflammatory cytokine concentration was performed on lung lysates using a multiplex an enzyme-linked immunosorbent assay (ELISA) for murine interleukin (IL)-1β, IL-6, tumour necrosis factor-alpha (TNF-α), monocyte chemoattractant protein (MCP)-1, macrophage inflammatory protein (MIP)-2, leukaemia inhibitory factor (LIF), granulocyte–macrophage colony-stimulating factor (GM-CSF), and regulated upon activation, normal T cell expressed and presumably secreted (RANTES) (Bio-Rad, CA, USA). The levels of cytokines were normalized against total protein concentration.

### Physiological measurements

At postnatal weeks 6, invasive plethysmography was performed using the flexiVent system (SCIREQ, Montreal, QC, Canada) as described before [23]. Changes to respiratory system resistance (Rrs), compliance (Crs), and pressure–volume loop (PV loop) were measured. Transthoracic echocardiography was also performed using a Vevo 2100 (VisualSonics, Toronto, Canada) as described previously [23]. Changes to the pulmonary artery acceleration time (PAT), the pulmonary artery ejection time (PET) and right ventricle anterior wall thickness (RVAWT) were measured.

### Statistical analysis

Data were expressed as mean ± standard error of mean (SEM). Statistical significance was determined with one-way analysis of variance (ANOVA) accompanied by the Bonferroni post hoc test for multiple groups or the Mann–Whitney test when comparing between two groups. Statistical significance was accorded when *p* < 0.05.

## Results

### Isolation and characterization of term and preterm hAEC-EVs

**Fig. 2:**
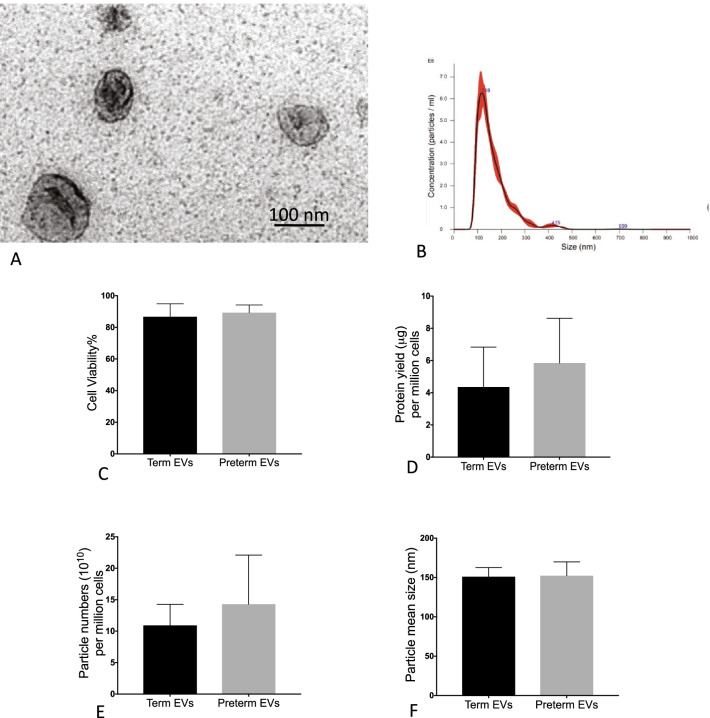
Characterization of human amnion epithelial cell-derived extracellular vesicle (hAEC-EV) (*n* = 5). **A** The representative transmission electron microscopy image of isolated hAEC-EVs. **B** The representative figure of particle distribution in hAEC-EVs. There was no significant difference in cell viability between term and preterm hAECs (**C**), and there were no differences in protein yield (**D**) particle numbers (**E**), and particle mean size (**F**) between term and preterm hAEC-EVs

hAEC-EVs typically exhibited a cup-shaped morphology across all cell lines regardless of gestational age of the donor (Fig. 2A). We did not observe significant differences in the cell viability, EV protein yield, EV particle number, particle mean size and size distribution between term and preterm hAECs (Fig. 2B–F).

**Fig. 3:**
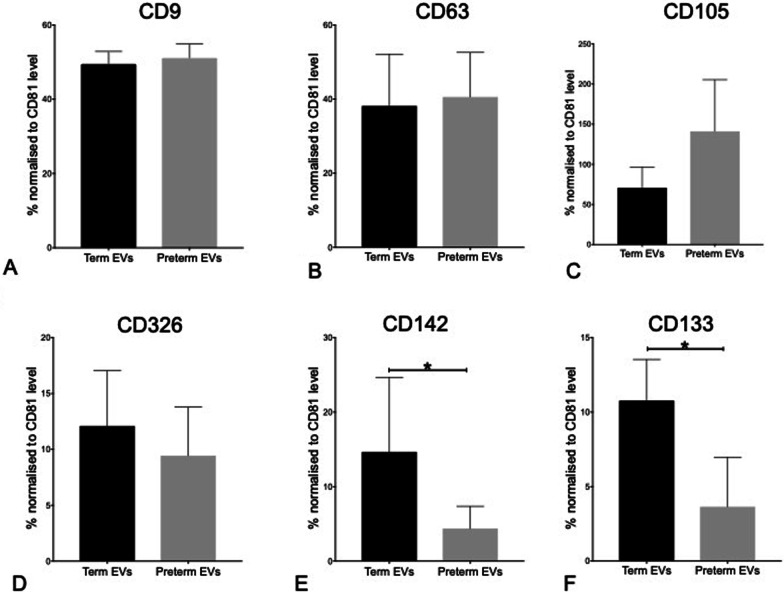
EV surface epitopes detected by MACSPlex (*n* = 5). All hAEC-EVs presented EV common tetraspanins CD9 (**A**) and CD63 (**B**), surface epitope CD105 (**C**), and epithelial marker CD326 (**D**). There were no differences in expression levels of these surface epitopes between term EVs and preterm EVs. Term EVs expressed higher levels of CD142 (**E**) and CD133 (**F**) compared to preterm EVs (*p < 0.05)

hAEC-EVs displayed tetraspanins common to EVs, namely CD9, CD81 and CD63, as well as soluble vascular endothelial growth factor (VEGF) receptor CD105 and epithelial cell marker CD326. There were no differences in expression levels of these surface epitopes between term and preterm hAEC-EVs (Fig. 3A–D). However, term hAEC-EVs displayed higher levels of CD142 and CD133 compared to preterm hAEC-EVs (Fig. 3E–F).

**Fig. 4:**
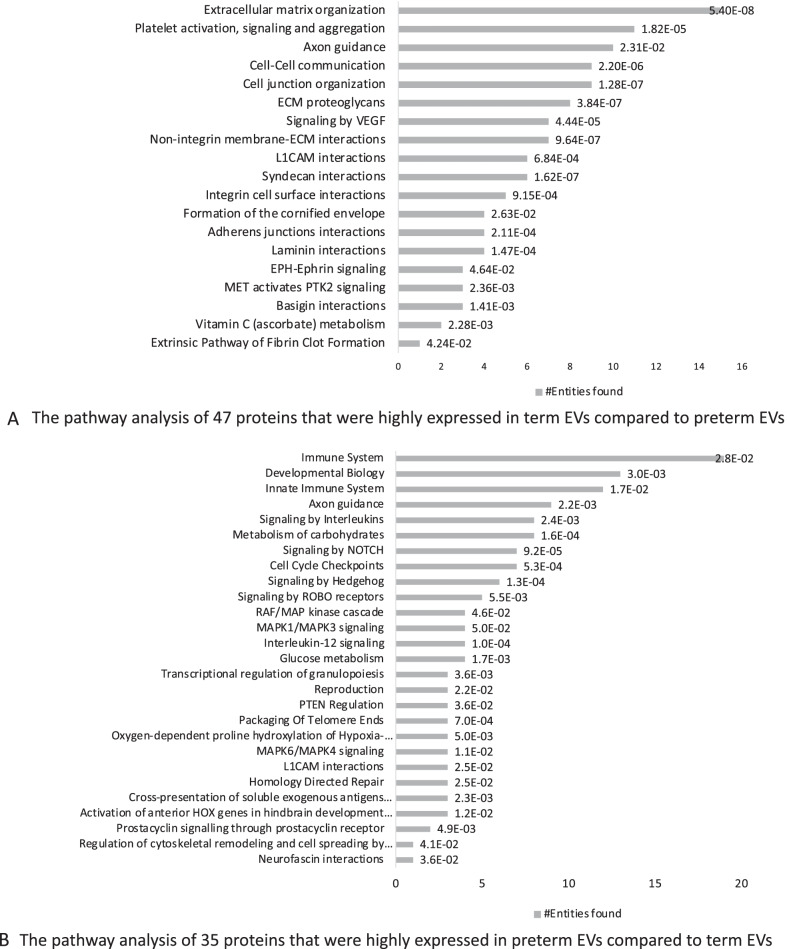
The pathway analysis of proteins. (*n* = 5). **A** The pathway analysis of 47 proteins that were highly expressed in term EVs compared to preterm EVs with fold change > 20. There were 19 pathways including extracellular organization (15 proteins related), cell junction organization (9 proteins related), integrin cell surface interaction (4 proteins related) and adherens junction interactions (4 proteins related). **B** The pathway analysis of 35 proteins that were highly expressed in preterm EVs compared to term EVs with fold change > 20. There were 27 pathways including immune system (13 proteins related), development biology (13 proteins related), innate immune system (12 proteins related) and signalling by Hedgehog (6 proteins related)

Proteomic analysis confirmed that term and preterm hAEC-EV samples contained EV tetraspanins such as CD9 and CD81, as well as other EV markers such as Alix, heat shock proteins (HSP) 70 and syntenin binding protein (SDCBP). EV-exclusion markers such as apolipoproteins (APO) A1, APOAB and albumin (ALB) were not detected in any hAEC-EV samples (data not shown). The 47 proteins were more than 20-fold higher in term hAEC-EVs (Table 1), while 35 proteins were more than 20-fold higher in preterm hAEC-EVs (Table 2). Pathway analysis of proteins given in Tables 1 and 2 is shown in Fig. 4A, B, respectively.

**Table 1:** 47 proteins with > 20-fold change in term hAEC-EVs compared to preterm hAEC-EVs

Protein names	Gene names	LogFC
Mucin-16	MUC16 CA125	26.3
Alpha-2-macroglobulin-like protein 1	A2ML1 CPAMD9	25.3
Collagen alpha-1(XVII) chain	COL17A1 BP180 BPAG2	25.3
SPARC	SPARC ON	25.2
Prostaglandin F2 receptor negative regulator	PTGFRN CD9P1 EWIF FPRP KIAA1436	25.0
Collagen alpha-1(XII) chain	COL12A1 COL12A1L	24.9
Syntenin-2	SDCBP2 SITAC18	24.8
Syntaxin-4	STX4 STX4A	24.8
Small proline-rich protein 3	SPRR3 SPRC	24.6
Sorcin	SRI	24.4
Monocarboxylate transporter 4	SLC16A3 MCT4	24.4
Synaptosomal-associated protein 23	SNAP23	24.1
F-actin-capping protein subunit alpha-1	CAPZA1	24.0
Serine protease 23	PRSS23 ZSIG13 UNQ270/PRO307	23.8
Unconventional myosin-Ic	MYO1C	23.7
Catenin beta-1	CTNNB1 CTNNB OK/SW-cl.35 PRO2286	23.7
Cystatin-M	CST6	23.6
Protein S100-A13	S100A13	23.5
Antithrombin-III	SERPINC1 AT3 PRO0309	23.5
Haemoglobin subunit beta	HBB	23.3
Syndecan-4	SDC4	23.2
Cytoplasmic FMR1-interacting protein 1	CYFIP1 KIAA0068	23.2
Radixin	RDX	23.1
Plasminogen activator inhibitor 1	SERPINE1 PAI1 PLANH1	23.0
Monocarboxylate transporter 1	SLC16A1 MCT1	23.0
N(G),N(G)-dimethylarginine dimethylaminohydrolase 2	DDAH2 DDAH G6A NG30	22.8
Choline transporter-like protein 1	SLC44A1 CD92 CDW92 CTL1	22.8
Tissue factor	F3	22.7
Catenin delta-1	CTNND1 KIAA0384	22.7
Gap junction beta-3 protein	GJB3 CX31	22.5
Tropomyosin alpha-1 chain	TPM1 C15orf13 TMSA	22.5
Glutathione S-transferase omega-1	GSTO1 GSTTLP28	22.4
Putative annexin A2-like protein	ANXA2P2 ANX2L2 ANX2P2 LPC2B	22.4
F-actin-capping protein subunit beta	CAPZB	22.3
Lysosome-associated membrane glycoprotein 1	LAMP1	22.3
Brain-specific angiogenesis inhibitor 1-associated protein 2	BAIAP2	22.1
Syntaxin-binding protein 3	STXBP3	21.8
Serine/threonine-protein phosphatase CPPED1	CPPED1 CSTP1	21.8
Plakophilin-2	PKP2	21.7
Src substrate cortactin	CTTN EMS1	21.7
Charged multivesicular body protein 4b	CHMP4B C20orf178 SHAX1	21.6
Protein piccolo	PCLO ACZ KIAA0559	21.5
Ras-related protein R-Ras	RRAS	21.4
Laminin subunit gamma-1	LAMC1 LAMB2	21.3
Guanine nucleotide-binding protein G(i) subunit alpha-1	GNAI1	21.3
Annexin A13	ANXA13 ANX13	20.6
Glutaredoxin-3	GLRX3 PICOT TXNL2 HUSSY-22	20.4

**Table 2:** 35 proteins with > 20-fold change in preterm hAEC-EVs compared to term hAEC-EVs

Protein names	Gene names	LogFC
Protein kinase C	PACSIN1 KIAA1379	26.9
Myosin light chain 3	MYL3	25.1
Myosin-6	MYH6 MYHCA	24.7
Histone H2AX	H2AFX H2AX	23.9
Barrier-to-autointegration factor	BANF1 BAF BCRG1	23.1
Malate dehydrogenase	MDH1 MDHA	22.9
Fructose-bisphosphate aldolase C	ALDOC ALDC	22.9
Complement factor H	CFH HF HF1 HF2	22.6
Heterogeneous nuclear ribonucleoproteins A2/B1	HNRNPA2B1 HNRPA2B1	22.4
Transaldolase	TALDO1 TAL TALDO TALDOR	22.4
Dihydropyrimidinase-related protein 2	DPYSL2 CRMP2 ULIP2	22.4
Glycogen phosphorylase, liver form	PYGL	22.3
ATP synthase subunit alpha	ATP5F1A ATP5A ATP5A1 ATP5AL2 ATPM	22.1
Rab GDP dissociation inhibitor alpha	GDI1 GDIL OPHN2 RABGDIA XAP4	22.1
Proteasome subunit alpha type-3	PSMA3 HC8 PSC8	22.0
E3 ubiquitin-protein ligase TRIP12	TRIP12 KIAA0045 ULF	22.0
Serine/arginine-rich splicing factor 2	SRSF2 SFRS2	21.9
1-phosphatidylinositol	PLCD1	21.8
Histone H1.0	H1F0 H1FV	21.8
60S acidic ribosomal protein P2	RPLP2 D11S2243E RPP2	21.5
60S acidic ribosomal protein P0	RPLP0	21.5
Malate dehydrogenase	MDH2	21.4
Guanine nucleotide-binding protein G(s) subunit alpha isoforms short	GNAS GNAS1 GSP	21.4
Transitional endoplasmic reticulum ATPase	VCP	21.3
Proteasome subunit alpha type-7	PSMA7 HSPC	21.2
Proteasome subunit alpha type-6	PSMA6 PROS27	21.1
Nuclease-sensitive element-binding protein 1	YBX1 NSEP1 YB1	20.9
Epithelial membrane protein 3	EMP3 YMP	20.8
Actin-related protein 2/3 complex subunit 4	ARPC4 ARC20	20.6
Dipeptidyl peptidase 4	DPP4 ADCP2 CD26	20.5
Syntenin-1	SDCBP MDA9 SYCL	20.5
Phosphoglucomutase-1	PGM1	20.3
Cytoplasmic dynein 1 intermediate chain 2	DYNC1I2 DNCI2 DNCIC2	20.2
Heterogeneous nuclear ribonucleoprotein D0	HNRNPD AUF1 HNRPD	20.0
Ubiquitin-like protein 3	PACSIN1 KIAA1380	20.0

### Comparative in vitro potency of term versus preterm hAEC-EVs

**Fig. 5:**
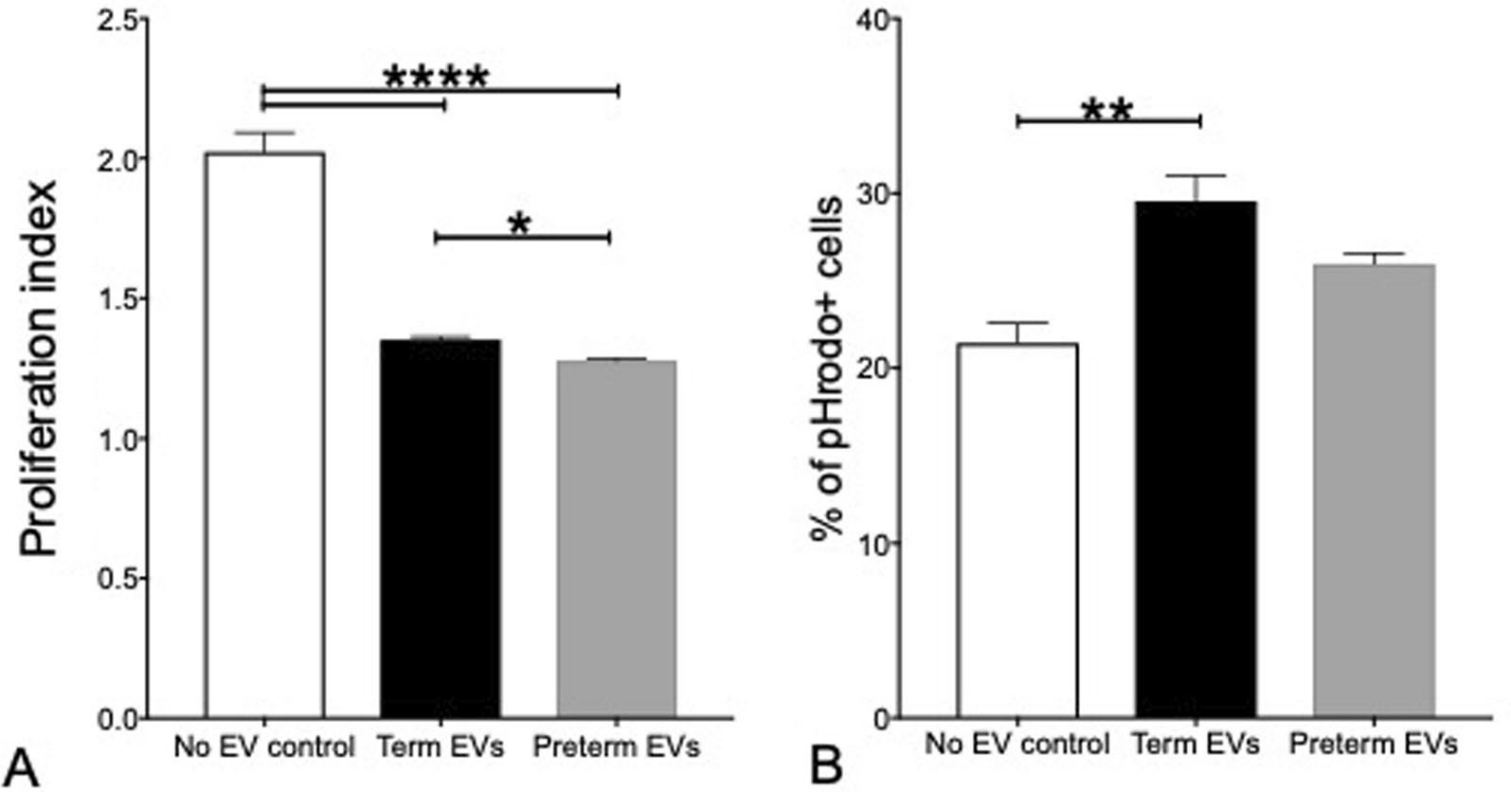
In vitro potency assays (*n* = 5). **A** Preterm EVs suppressed T cell proliferation more significantly than term EVs with lower proliferation index. **B** Term EVs improved macrophage phagocytosis more significantly than preterm EVs. (****p < 0.0001, **p < 0.01, *p < 0.05)

The ability of term and preterm hAEC-EVs to directly influence the behaviour of T cells and macrophages was assessed. Here, we observed that while both term and preterm hAEC-EVs suppressed T cell proliferation, greater suppression was achieved with preterm hAEC-EVs (Fig. 5A). In contrast, term hAEC-EVs, but not preterm hAEC-EVs, significantly increased macrophage phagocytosis (Fig. 5B). These findings suggest that differences in EV cargo may be related to differential effects on target cells.

### Term hAEC-EVs ameliorated alveolar simplification

**Fig. 6:**
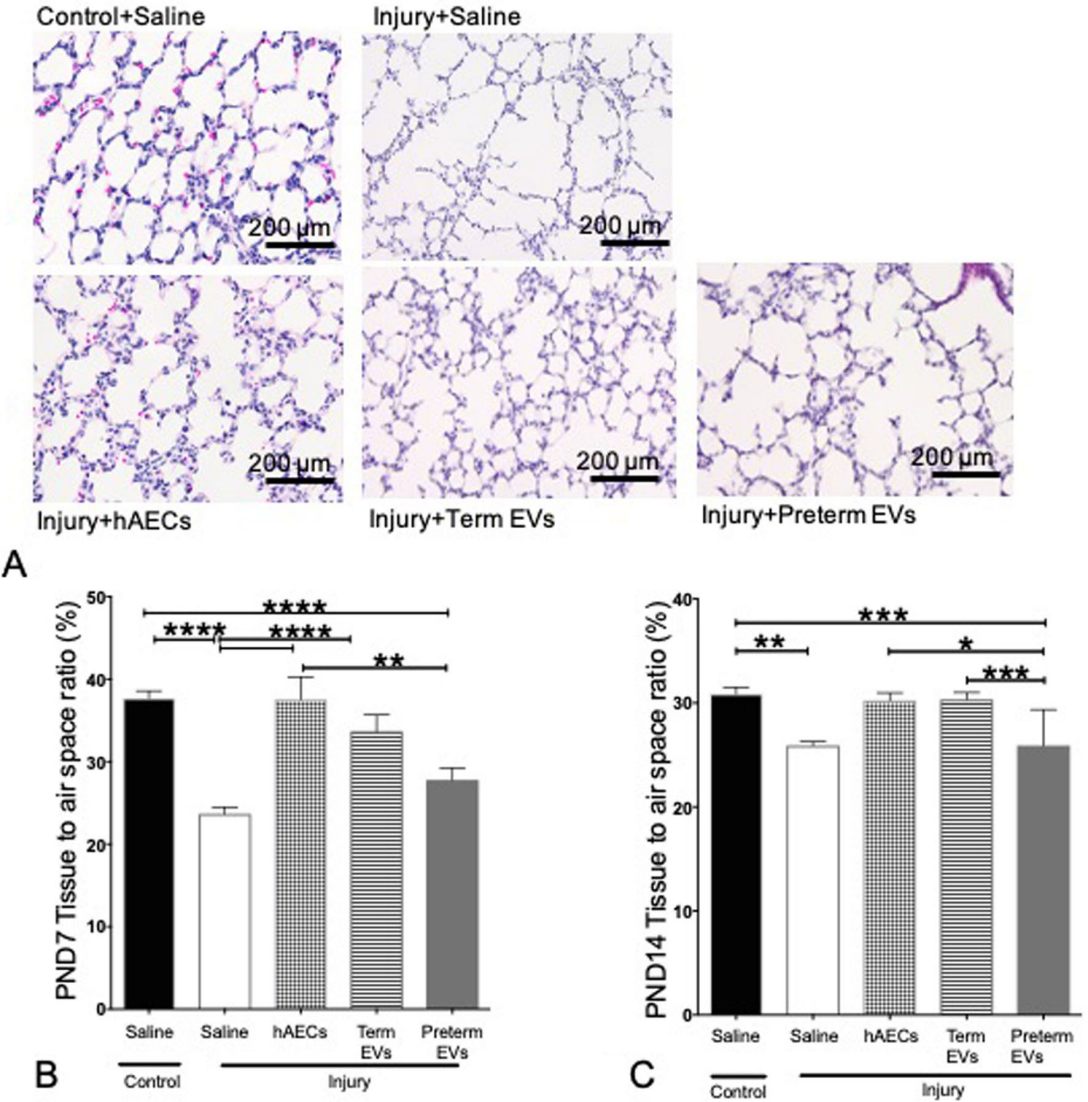
Tissue-to-air space ratio on PND 7 and 14 (*n* = 6). **A** Representative images for H&E staining (PND7). **B** On PND7, tissue-to-air space ratio was decreased in saline-treated injured group compared to control groups. In contrast to hAEC treatment, only term but not preterm EVs improved lung structure. **C** On PND14, tissue-to-air space ratio remained lower in saline-treated injured group. Term EV treatment mitigated tissue-to-air space ratio and made it comparable to hAEC treatment group and healthy control group. However, preterm EVs did not improve lung structure. (*p < 0.05, **p < 0.01, ***p < 0.001, ****p < 0.0001)

The combination of intra-amniotic LPS and neonatal hyperoxia significantly reduced tissue-to-air space ratio by PND 7 and 14 (*p* < 0.001 and *p* < 0.01) compared to control groups (Fig. 6A–C). Only term hAEC-EVs improved tissue-to-air space ratio such that they were not significantly different to the control group and hAEC treatment group by PND7 (Fig. 6B). Furthermore, term hAEC-EVs mitigated the injury by PND14 as seen by the improvement in tissue-to-air space ratio, such that they were comparable to the control group (Fig. 6A, C). In contrast, tissue-to-air space ratio of animals given preterm hAEC-EVs remained significantly lower than healthy controls (*p* < 0.001, Fig. 6A, C). The tissue-to-airspace ratio achieved by the hAEC was higher than both hAEC-EV treatment groups by PND7 (*p* < 0.05). The outcomes achieved by either hAECs or term hAEC-EVs were comparable by PND14 (Fig. 6B, C).

### Term hAEC-EV treatment improved secondary septal crest density

**Fig. 7:**
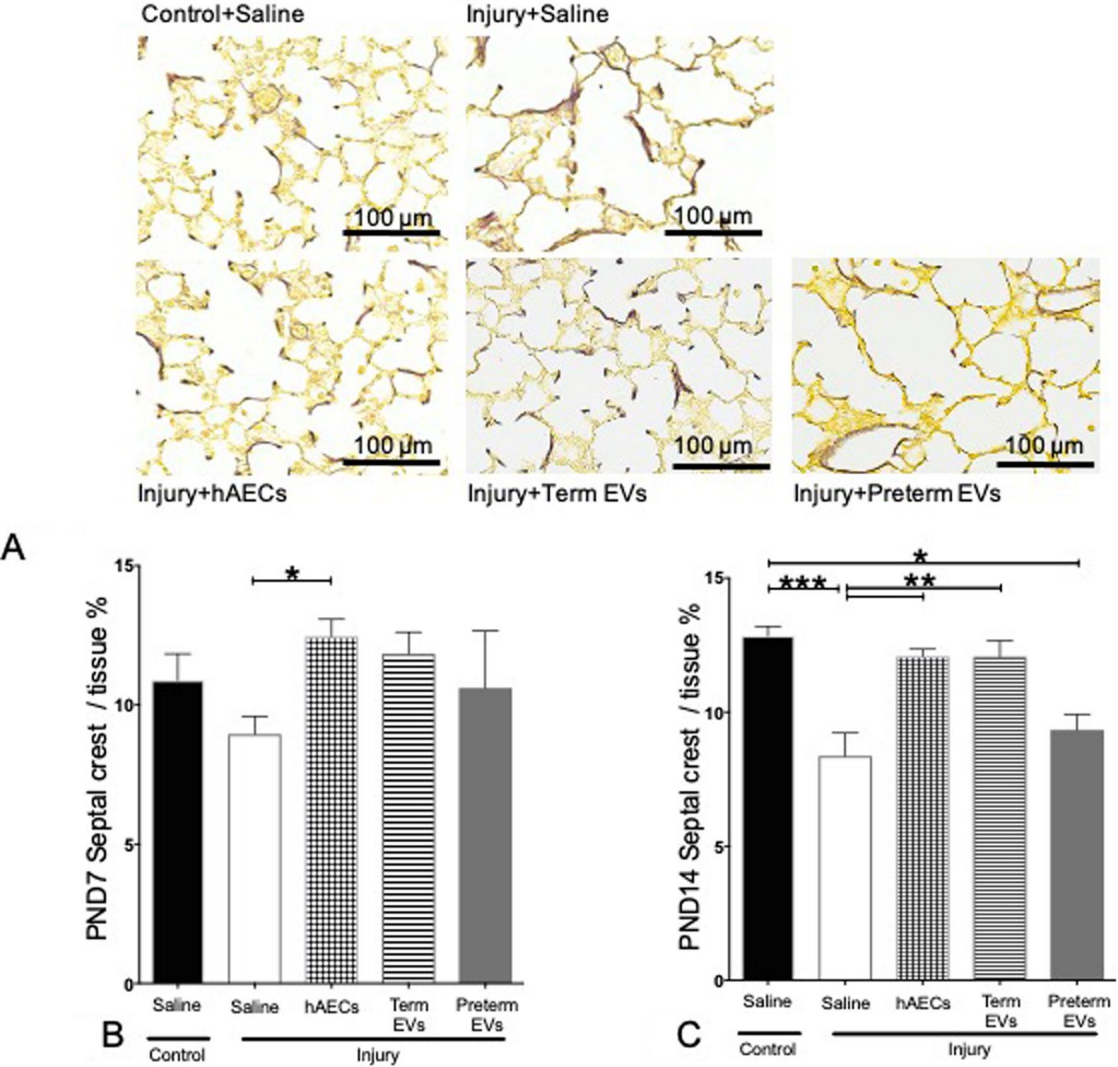
Hart’s staining on PND7 and PND14 (*n* = 6). **A** Representative images for Hart’s staining on PND14. **B** Term hAEC injection improved secondary septal crest density to control levels. **C** Secondary septal crest density was decreased in the saline-treated injured group, hAEC and term EV injection normalized it to control levels. However, preterm EV treatment did not improve secondary septal crest density. (*p < 0.05, **p < 0.01, ***p < 0.001)

Our model of experimentally induced BPD caused a decrease in septal crest density at PND7 and PND14 compared to control animals (Fig. 7A–C). hAEC treatment in BPD animals significantly increased secondary septal crest density at PND7 and PND14, such that they were comparable to control (Fig. 7B, C). Term but not preterm hAEC-EVs also increased septal crest density but only statistically significant by PND14 compared to BPD animals (Fig. 7C).

### Term hAEC-EV treatment decreased lung inflammation

**Fig. 8:**
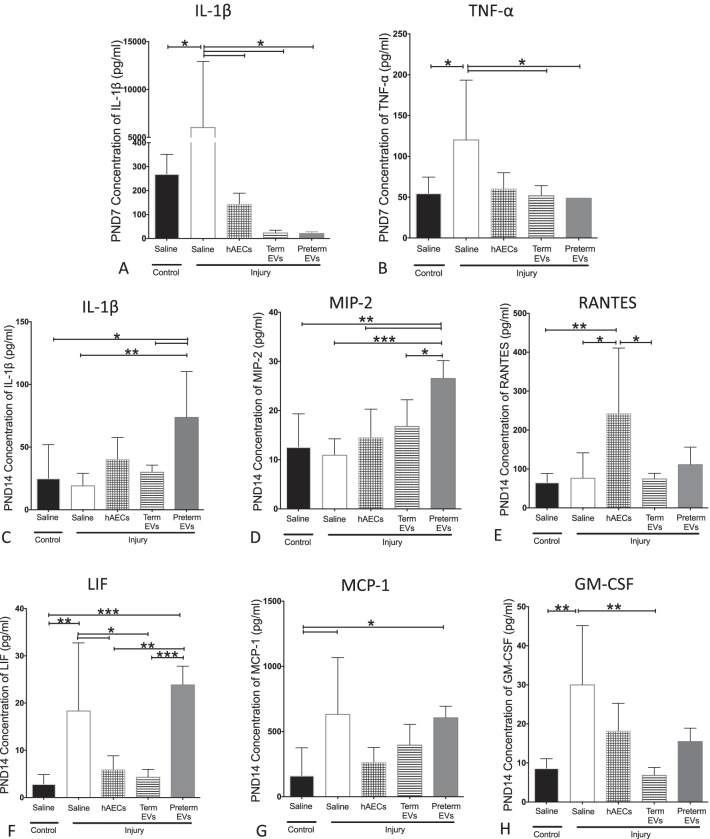
Changes of inflammatory cytokine levels in mouse lung lysate (*n* = 6). On PND7, IL-1β (**A**) and TNF-α (**B**) levels were increased in injured group, and EVs normalized them to control levels, which is comparable to hAEC treatment. On PND14, IL-1β (**C**) and MIP-2 (**D**) levels were higher in preterm EV-treated group. **E** Levels of RANTES only increased in the hAEC treatment group, but not in the EV group. LIF (**F**), MCP-1 (**G**) and GM-CSF (**H**) levels were increased in the injured group, and both hAECs and term EVs reduced them to control levels. However, levels of LIF and MCP-1 remained higher in the preterm EV treatment group. (*p < 0.05, **p < 0.01, ***p < 0.001)

By PND7, both term and preterm hAEC-EV administration reduced IL-1β and TNF-α to control levels (*p* < 0.05, Fig. 8A, B), which was comparable to hAEC treatment. Interestingly, higher levels of IL-1β and MIP-2 were observed in the group treated with preterm hAEC-EVs (Fig. 8C, D). The level of RANTES was elevated in hAEC treatment group, but not in the term hAEC-EV group (*p* < 0.05, *p* < 0.01, Fig. 8E). The levels of LIF, MCP-1 and GM-CSF were higher in the injury group. Both hAECs and term hAEC-EVs reduced them to control levels, but the levels of LIF and MCP-1 remained high in preterm hAEC-EV-treated mice (*p < 0.05, **p < 0.01, ***p < 0.001, Fig. 8F–H). Levels of TNF-α and IL-6 were below the limit of detection.

### Term hAEC-EV treatment induced type ii alveolar cell but not bronchioalveolar stem cell proliferation

**Fig. 9:**
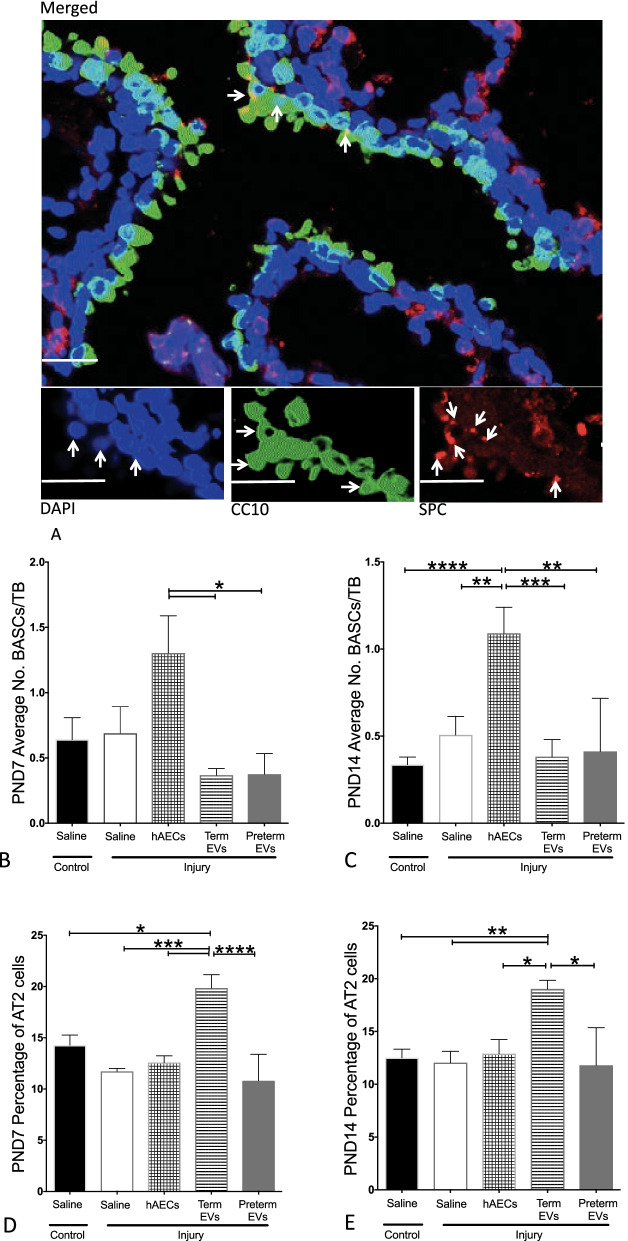
Bronchioalveolar stem cells (BASCS) and type II alveolar cells (AT2s) in mouse lungs at PND7 and PND14 (*n* = 6). **A** Representative image of pro-SPC (red) and CC10 (green) immunohistochemical staining on mouse lung tissues where arrows point to BASCs (scale bars = 50 μm). **B**, **C** The average number of BASCs per terminal bronchiole on PND7 (**B**) and PND14 (**C**). In contrast to hAEC treatment group, where the average number of BASCs was significantly higher than control group on PND14, it did not change in EV groups on both time points. **D**, **E** The percentage of AT2 cells was significantly increased in term EV treatment group on both PND7 (**D**) and 14 (**E**) compared to control group, which is on contrast to the unchanged AT2 cell percentage in both hAEC and preterm EV treatment groups (*p < 0.05, **p < 0.01, ***p < 0.001, ****p < 0.0001)

Bronchioalveolar stem cells (BASCs) express both pro-SPC and CC10. They are located at the terminal bronchioles. AT2s stained positive for pro-SPC and are located throughout the parenchyma (Fig. 9A). Experimentally induced BPD had no effect on BASC and AT2 cell activation. hAEC treatment significantly increased the average number of BASCs per terminal bronchiole compared to BPD and control animals by PND14. However, the average number of BASCs per terminal bronchiole did not change in response to either hAEC-EV group on both PND7 and PND14 (Fig. 9B, C). However, the percentage of AT2 cells was significantly increased in the term hAEC-EV treatment group at both time points compared to control group (*p* < 0.05 and *p* < 0.01). In contrast, there was no significant change in either preterm hAEC-EV or hAEC treatment groups (Fig. 9D, E).

### Term hAEC-EV treatment increased the number of vWF-positive pulmonary vessels

**Fig. 10:**
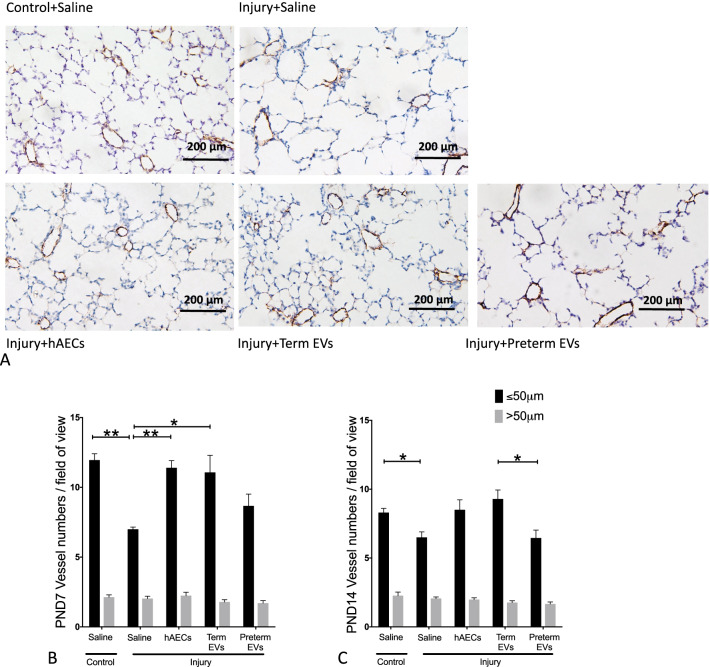
von Willebrand factor (vWF) immunohistochemistry in mouse lung tissue at PND 7 and 14 (*n* = 6). **A** Representative images for vWF staining on PND14. **B**, **C** The average number of vessels per field of view on PND7 (**B**) and PND14 (**C**). The average number of vessels with diameter ≤ 50 µm (black bars) was decreased in saline-treated injured mice, which was restored after hAEC and term EV treatment on both PND7 and PND14. There was no difference in the numbers of larger blood vessels (> 50 μm, grey bars) between groups. (*p < 0.05, ** *p* < 0.01)

The number of small pulmonary vessels (≤ 50 µm in diameter) was significantly decreased in the BPD group compared to controls on both PND7 and PND14 (*p* < 0.01 and *p* < 0.05, Fig. 10A–C). hAEC and term hAEC-EV treatment restored pulmonary vascularization to control levels, but this was not achieved by preterm hAEC-EV treatment. (Fig. 10B, C). Larger blood vessels (> 50 µm) were unaffected across all experimental groups.

### Term hAEC-EV treatment reduced peripheral pulmonary arterial remodelling

**Fig. 11:**
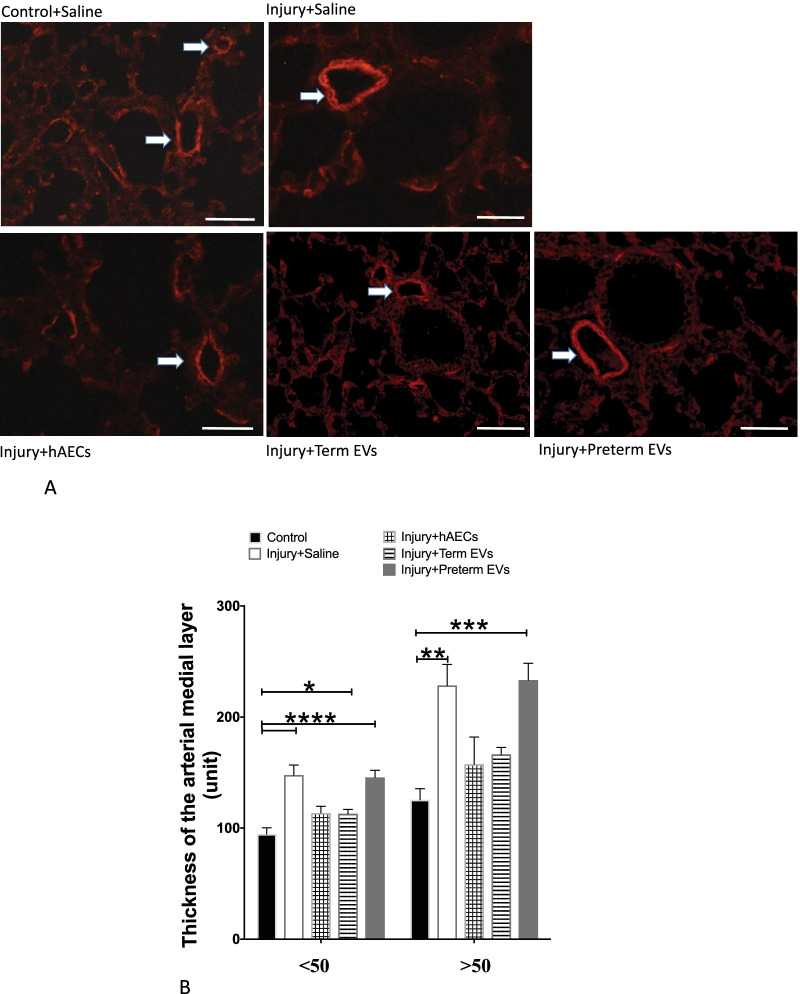
Smooth muscle alpha-actin (α-SMA) immunofluorescence in mouse lung tissue at PND14 (*n* = 6). **A** Representative images for α-SMA immunofluorescence in mouse lung tissues at PND14. Scale bars = 50 μm. Vessels are indicated with white arrows. **B** By PND 14, arterial medial thickness was increased in injured mice. This was mitigated by both hAEC and term EV treatment, but not preterm EV treatment. (*p < 0.05, **p < 0.01, ***p < 0.001, ****p < 0.0001)

Pulmonary arterial hypertension caused by vascular remodelling is a common secondary complication to BPD. In order to assess this in experimentally induced BPD, the medial layer of the pulmonary arteries was stained for α-SMA (Fig. 11A). The thickness of the arterial medial layer was significantly increased in the experimental BPD group by PND14, but this was attenuated in the hAEC and term hAEC-EV treatment groups (*p* < 0.01, *p* < 0.001, *p* < 0.0001, Fig. 11B).

### Lung tissue-to-airspace ratio

**Fig. 12:**
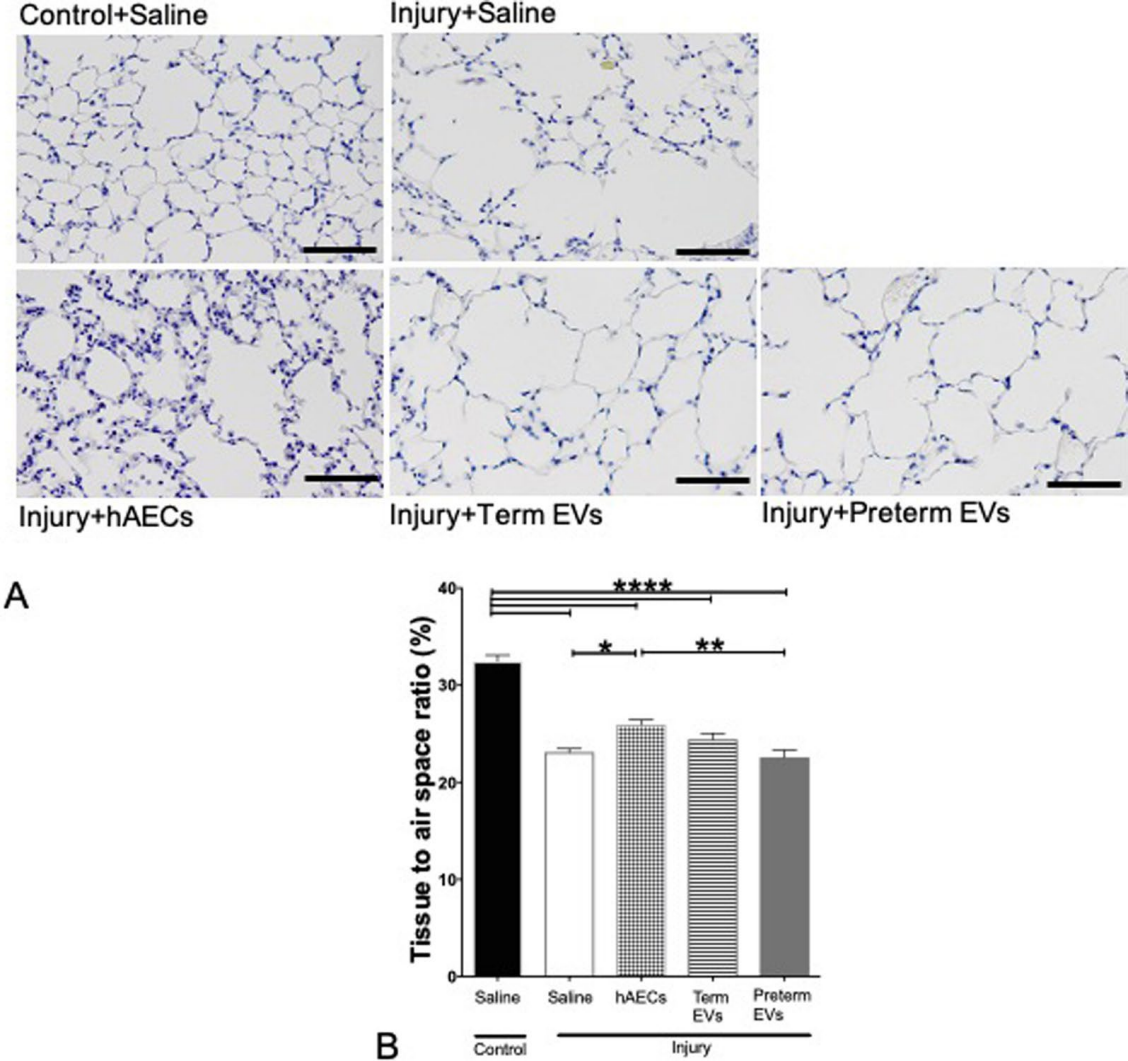
Hematoxylin and eosin (H&E) staining of mouse lung tissue at week 6 (*n* = 6). **A** The representative images for H&E staining on mouse lung tissues by week 6. Scale bars = 200 μm. **B** hAEC treatment improved the tissue-to-air space ratio compared to saline-treated injured mice, but remained lower than controls. Term EV treatment improved the tissue-to-air space ratio, making it between hAEC treatment group and saline-treated group, while preterm EV treatment made no significant difference to the saline-treated injured mice. (*p < 0.05, **p < 0.01, **** *p* < 0.0001)

We observed that the reduced tissue-to-airspace ratio persisted until week 6 but was significantly improved by hAEC treatment. Neither term nor preterm hAEC-EVs significantly improved tissue-to-airspace ratio by Week 6 (Fig. 12A, B).

### Term hAEC-EV treatment prevented the increase in airway responsiveness

**Fig. 13:**
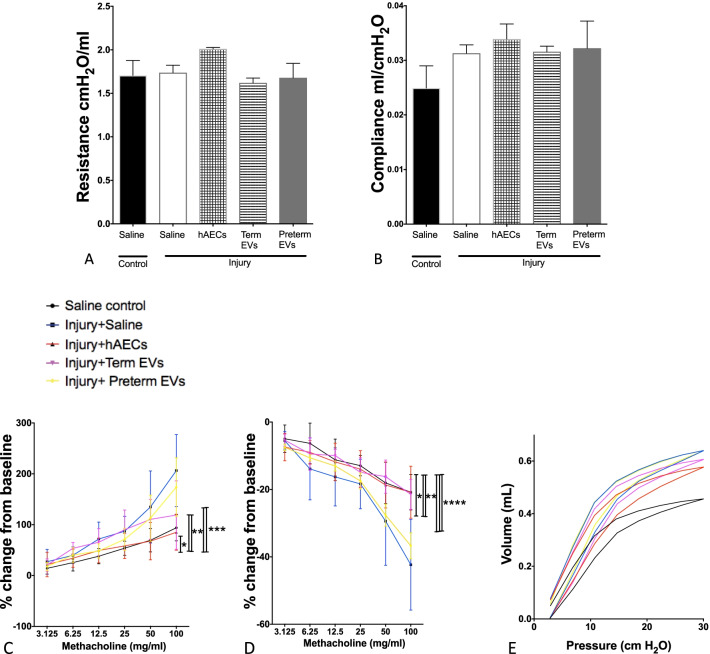
Invasive lung function test on 6-week-old mice (*n* = 6). **A**, **B** There were no significant differences in the baseline of either respiratory resistance (Rrs, **A**) or compliance (Crs, **B**) between groups. **C**–**D** Compared to control group, Rrs was significantly increased (**C**) and Crs was significantly decreased (**D**) at the 100 mg/ml methacholine dose in the injured group, but this effect was diminished with hAEC treatment and also improved with term EV treatment groups. (**E**) Pressure volume loop (PV loop) on week 6. There was a significant upward shift of the PV loop in the injured group compared to the control group. While the PV loop of both hAEC treatment group and term EV treatment group was intermediate to both control and saline-treated injured groups, preterm EV treatment had no effect on the position of the PV loop. (*p < 0.05, **p < 0.01, ***p < 0.001, ****p < 0.0001)

Lung function test at postnatal week 6 showed that baseline respiratory system resistance (Rrs) and compliance (Crs) did not significantly change between groups (Fig. 13A, B). When challenged with 100 mg/mL methacholine, the injured mice showed significantly increased Rrs and decreased Crs compared to the control (*p* < 0.001 and *p* < 0.0001, respectively, Fig. 13C, D). hAEC treatment restored Rrs and Crs to control levels. Term hAEC-EV treatment increased Crs to healthy mice level and decreased Rrs to the level that was between control and injured groups (*p* < 0.05, Fig. 13C, D). However, preterm hAEC-EVs did not mitigate airway response. The pressure–volume (PV) loop is generated by changes to lung volume during a respiratory cycle. Compared to control, the PV loop of the injured group saw a significant upward shift, indicating increased lung compliance and suggestive of reduced tissue elasticity. Both hAEC and term hAEC-EV treatment returned the loop downwards such that the PV loop sat between the control and injured groups. The PV loop position of mice that received preterm hAEC-EVs remained higher than control. (Fig. 13E).

#### Term hAEC-EV treatment prevented pulmonary hypertension and right ventricular hypertrophy

**Fig. 14:**
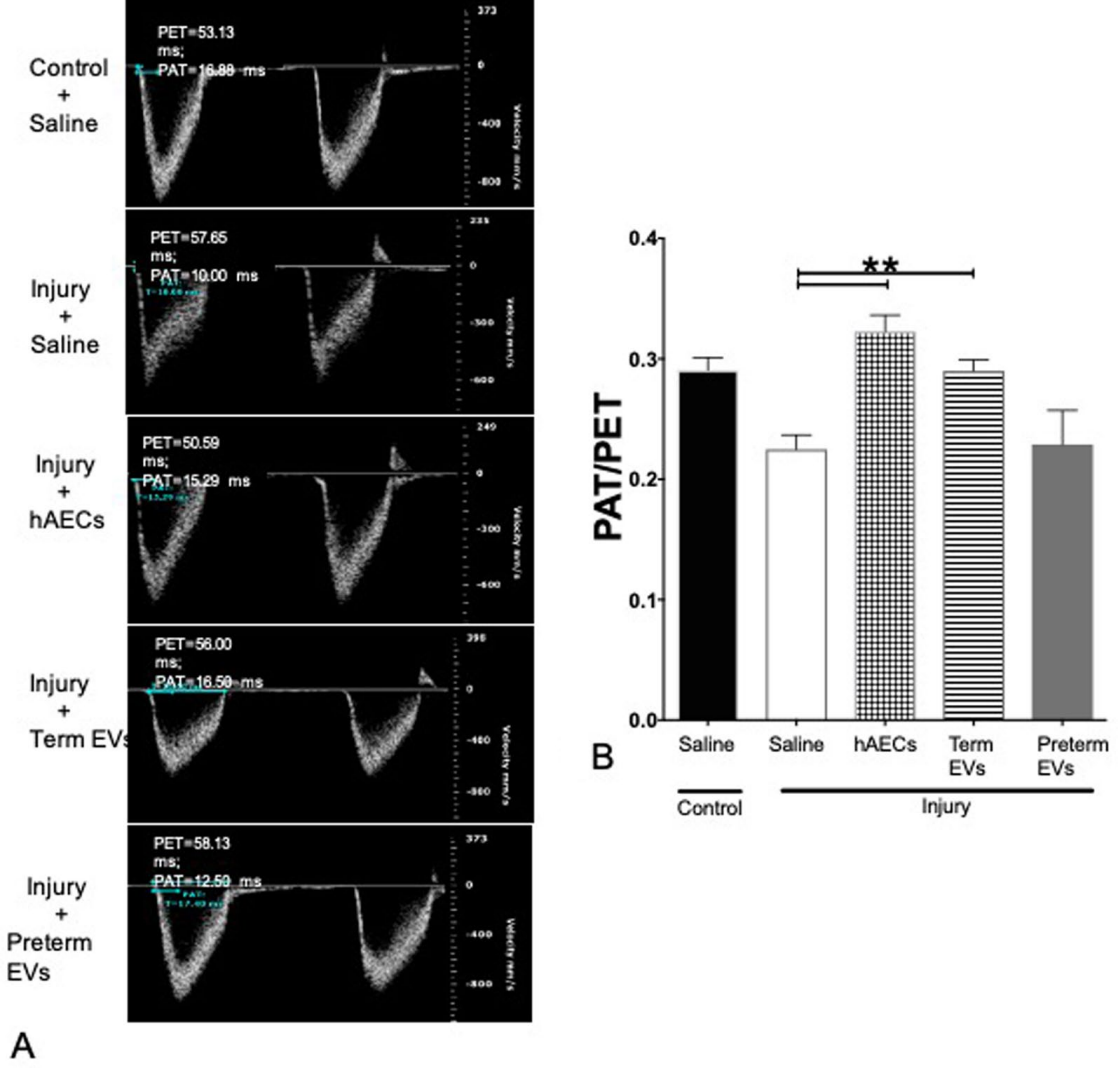
Changes in pulmonary artery flow on 6-week-old mice (*n* = 6). (**A**) Representative images of pulmonary artery flow. (**B**) Changes of PAT/PET (pulmonary artery acceleration time/ejection time) ratio. Injury decreased the ratio of PAT/PET, which indicated the development of pulmonary hypertension. Both hAEC treatment and term EV treatment restored the ratio back to control level; however, preterm EV treatment group did not have significant changes. (**p < 0.01)

**Fig. 15:**
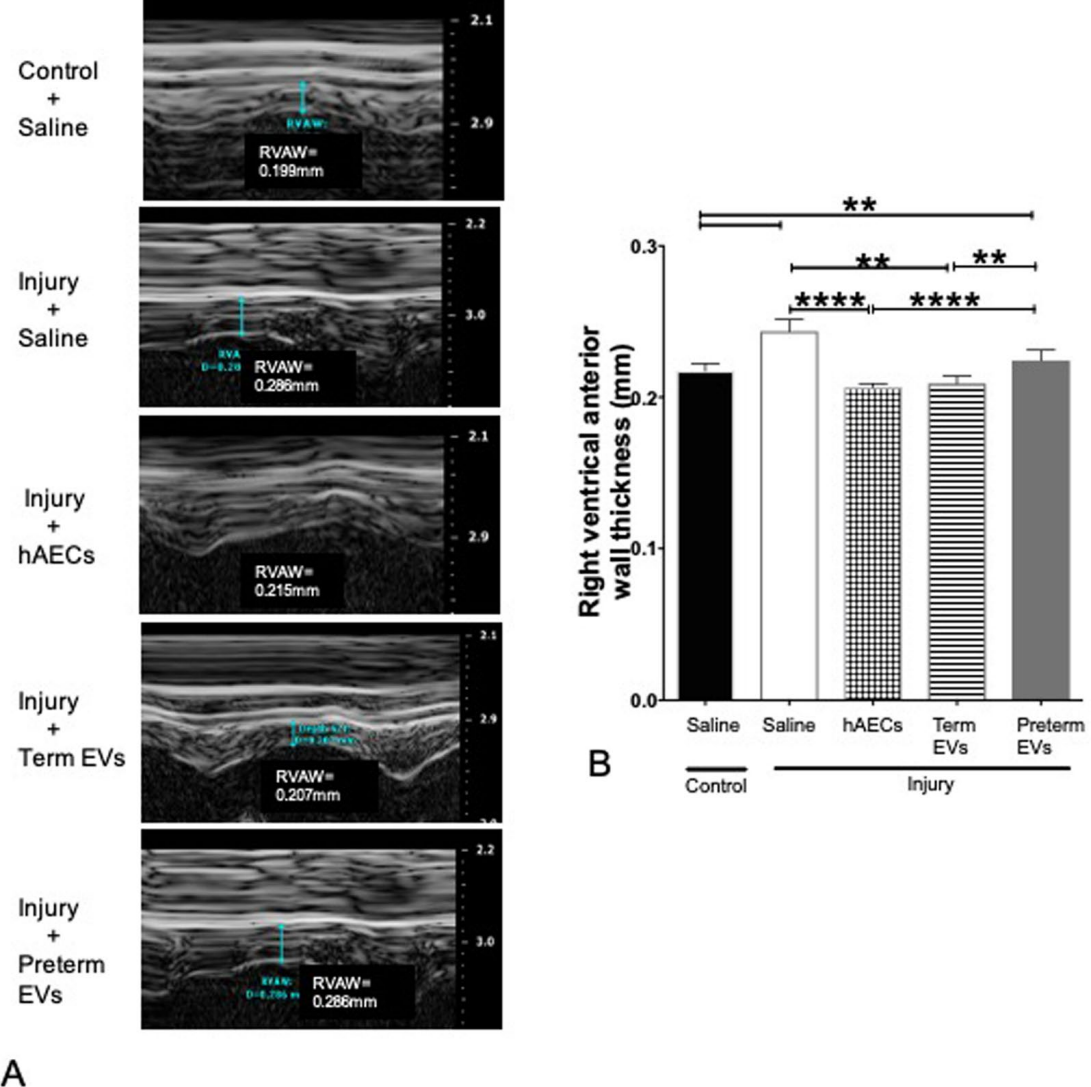
Changes to right ventricle anterior wall thickness (RVAWT) on 6-week-old mice (*n* = 6). (**A**) Representative images of the RVAWT. (**B**) The RVAWT increased in the injured mice, while both hAEC treatment and term EV treatment groups, but not preterm EV group, decreased the wall thickness to control levels. (**p < 0.01, ****p < 0.0001)

Echocardiography showed that at postnatal week 6, the injury group developed pulmonary hypertension with reduced pulmonary artery acceleration to ejection time (PAT/PET, Fig. 14A, B) and right ventricle hypertrophy with thickened RVAW (Fig. 15A, B). These were attenuated by hAEC and term hAEC-EV treatment but not preterm hAEC-EV treatment (Figs. 14A, B, 15A, B).

## Discussion

In this manuscript, we characterized term and preterm hAEC-EVs and assessed their efficacy in an experimentally induced BPD mouse model. This study provides the first evidence that hAEC-EVs from term and preterm pregnancy bore exosome characteristics based on size distribution, morphology and surface markers. We did, however, observe that EV cargo and functional potency between term hAEC-EVs and preterm hAEC-EVs were significantly different. Only term but not preterm hAEC-EVs were beneficial in the setting of BPD-like lung disease, which was comparable to the efficacy of term hAECs.

Both term and preterm hAEC-EVs appeared to be of the size range most commonly associated with exosomes, displaying their typical cup-shaped morphology with no significant difference in EV yield. In order to verify their identity, we sought to determine the presence of typical EV markers CD9, CD63, CD81, and ALIX. In the current study, we observed EV yield from hAECs with 2 to 9 μg EVs from 1 × 10^6^ cells, or 4 × 10^10^ to 24 × 10^10^ particles between different hAEC donors, which may be due to the human biological variability. However, there was no significant difference in protein yield, particle concentration and particle size distribution between term and preterm hAEC-EVs. It is worth noting that others have reported yields of 10 μg EVs from 2.5 × 10^5^ to 1 × 10^7^ human bone marrow-derived MSCs [26, 27].

Further, when we examined exosomal surface epitopes, we have shown that term hAEC-EVs had higher level of CD142 and CD133. CD142 is also called tissue factor (TF), which belongs to the cytokine receptor class II family, and it is known to exist with membrane vesicles [28]. CD142 was proven to induce pro-angiogenic matrix for blood vessel infiltration, and the activation of TF/VIIa (coagulation factor VIIa) signalling pathway promoted angiogenesis [29]. CD133 is one of the key biomarkers for isolation and characterization of stem cells, and it can be found in a few cells including epithelial cell membrane [30]. CD133^+^ cells are known to have regenerative properties including promoting cell differentiation and enhancing angiogenesis [31, 32] and have been used in a few clinical trials such as ischemia and hepatic fibrosis [33, 34]. Similarly found, MSC-derived EV containing CD133 epitope promoted recovery in a moue model of ischemia reperfusion renal injury, proving that EV therapeutics might be superior to cell-based therapy in terms of safety and versatility [35].

We demonstrated that the term hAEC-EVs, but not preterm hAEC-EVs, were efficacious in mitigating lung injury in a mouse model of experimental BPD. Specifically, we observed an increased tissue-to-air space ratio, secondary septal crest density and small pulmonary vessel numbers, suggesting that term hAEC-EVs may protect against the classical alveolar simplification associated with postnatal hyperoxia. This improvement in lung structure was accompanied by a reduction in lung inflammation with specific changes in IL-1β, TNF-α, LIF, MCP-1, MIP-2 and GM-CSF levels. Similar to term hAEC treatment, term hAEC-EV administration prevented peripheral pulmonary artery muscularization and further prevented pulmonary hypertension and right ventricle hypertrophy and improved lung function as a long-term outcome. Additionally, term hAEC-EV treatment was associated with increased numbers of AT2 cells, but without affecting the BASC population. This suggests that term hAEC-EVs may improve lung tissue regeneration either through stimulating the local lung stem cell AT2 niche or promoting the maturation of BASCs to AT2 cells.

On PND 7 and 14, the tissue-to-air space ratio and secondary septal crest percentage were significantly decreased in the injured group compared to the control group, and we observed that both term hAEC and term hAEC-EV treatment brought the percentages back to healthy levels. As angiogenesis promotes normal lung alveolar development and contributes to the maintenance of alveolar structure [36], BPD animals with simplified lung structure had fewer pulmonary vessels. Our previous studies showed that only term but not preterm hAECs-derived conditioned media supported vascular tubule formation [25]. This supports the findings in our current study where only term hAECs and term hAEC-EVs protected against the loss of small pulmonary vessels.

When observing another characteristic in BPD, lung inflammation, we found that term hAEC-EVs exerted anti-inflammatory effects by PND7. The elevated levels of IL-1β and TNF-α caused by the injury were diminished by either term hAECs, term hAEC-EVs or preterm hAEC-EVs. IL-1β and TNF-α are mainly secreted from activated macrophages and monocytes [37], which we had previously shown to be reduced by hAEC [23]. It is thus possible that term EVs may also modulate macrophage function. Along similar lines, MSC-derived EVs reportedly polarized macrophages from M1 to M2 phenotype *in vitro* [38], suppressed macrophage pulmonary infiltration in response to hypoxic lung injury [39] and attenuated burn-induced inflammation through reducing the expression of IL-1β and TNF-α in mice [40]. In addition, we also found in vitro that preterm hAEC-EVs suppressed T cell proliferation more significantly than term hAEC-EVs and that term hAEC-EVs improved macrophage phagocytosis more significantly than preterm hAEC-EVs. Similarly, MSC-derived EVs have also been shown to restore T cell function disrupted by neonatal hypoxia [41]. These data suggest that impact may be due to direct effect of EV cargo on macrophages.

The cytokine levels of GM-CSF, MCP-1, MIP-2 and LIF were significantly higher in injured animals by PND14, as previously reported [23]. Macrophages and monocytes are the major cell types secreting MIP-2 (IL-8 in human) and MCP-1 during immune response. MCP-1 regulates monocyte migration and infiltration and recruits and directs macrophage movement [42], and MIP-2 is a neutrophil chemoattractant [43]. It has been reported that alveolar macrophages secrete MIP-2 and MCP-1 during lung inflammation [44, 45], and elevated levels of MCP-1 and IL-8 were reported in lavage fluid of BPD-affected infants [46]. Additionally, GM-CSF is a known macrophage activator [47], and LIF is known to potentiate macrophage aggregation and activation [48]. In this study, we found that similar to term hAEC treatment, term hAEC-EVs reduced the above cytokines to control levels. It is likely that term hAEC-EVs reduced lung inflammation through modulating macrophage and neutrophil populations as we [20] and others [49, 50] have shown previously. Unexpectedly, preterm hAEC-EV treatment increased levels of IL-1β, MIP-2 and LIF compared to control animals. We are unsure of how preterm hAEC-EVs are increasing these factors. However, IL-1β, MIP-2 and LIF are produced by macrophages, so it could be that the preterm hAEC-EVs had a very specific impact on monocyte/macrophage recruitment and/or polarization which resulted in this unexpected increase.

In this study, we found that the percentage of BASC or AT2 cells was unchanged in the injured group. This correlates with previous studies that have shown that hyperoxia at 75% oxygen had no effect on BASC proliferation [51]. Surprisingly, unlike hAECs, term hAEC-EVs had no effect on BASC numbers, but significantly increased the percentage of AT2 on PND7 and PND14. The increase in AT2 correlated with the improvement in lung structure, which may have resulted in activation, proliferation and differentiation into AT1 to improve tissue-to-air space ratio. Some researchers have suggested that there are different mechanisms involved in AT2 and BASC proliferation and differentiation [52–55]. The observation that term hAEC-EVs increased AT2 numbers while term hAECs increased BASC numbers could therefore be attributed to differential activation of signalling pathways. However, neither BASC nor AT2 proliferation was observed in preterm hAEC-EV-treated group.

In our experimentally induced BPD animals, we observed thickened and muscularized pulmonary vessels on PND14, pulmonary hypertension, right ventricle hypertrophy, and declined lung function with increased resistance and increased compliance. Similar to the term hAEC administration, term hAEC-EVs prevented early pulmonary vascular muscularization and later on secondary pulmonary hypertension. Kourembanas and her colleagues induced BPD injury in mice by exposing neonates to 75% oxygen for a week; they also observed pulmonary vascular remodelling, increased lung capacity and elevated right ventricular systolic pressure, and found that both bone marrow-derived MSCs (BM-MSC) and Wharton’s jelly MSCs (WJ-MSCs)-derived EVs ameliorated vascular remodelling and improved lung function [6, 56]. It is worth noting that although EVs do not package all the factors that cells contain, this study implies that term hAEC-EVs may carry sufficient bioactive material to exert reparative effects. This is supported by studies that showed MSC-EVs had reparative effects, but EV-depleted MSC conditioned media had no effect in a few preclinical models including hypoxic-induced pulmonary hypertension and cardiotoxin-induced muscle injury [39, 57]. From what mentioned above, Kourembanas and her colleagues treated BPD mice with EVs derived from different sources of MSCs (BM-MSCs and WJ-MSCs) and found both achieved the same therapeutic outcomes. This suggested that EVs from different cell sources could transport effective cargo to exert efficacy. Future experiments targeting specific cargo like microRNA (miRNA) have shown enormous promise for the development of therapeutic agents for human diseases like BPD [58, 59].

Proteomic analysis revealed that EV cargo from term and preterm hAECs was significantly different. Proteins that were more highly expressed in term hAEC-EVs were enriched in several pathways associated with functional characteristics observed in our current study. The extracellular matrix (ECM) organization pathway is crucial to stem cell lineage specification, and cell migration and proliferation through its dynamic regulation of the microenvironment [60]. Cell junction organization pathway and adherens junction interactions pathway are cell–cell or cell–ECM contacts that are required for cell survival, differentiation, and migration [61]. The ECM homeostasis is important in normal lung development, and collagen is one of the major compositions of the ECM [62, 63]. Increased gene expression level of collagen alpha-1 (cola1) was detected in neonatal mice and aided the process of alveolar development [64]. Integrin-mediated cell–ECM interactions are known to play an important role in normal lung development too, and higher expression levels of integrin alpha-1, 2, 6 and beta1 were reported in the bronchial and alveolar epithelium during the alveolar stage of lung development [65, 66]. Indeed, cola1 and integrin alpha6 were highly expressed in term hAEC-EVs compared to preterm hAEC-EVs. Among the proteins that were significantly higher in term hAEC-EVs, 15 proteins were associated with extracellular matrix organization, 9 proteins were associated with cell junction organization and 5 proteins were associated with integrin cell surface interactions. This indicated that term hAEC-EVs may support normal lung development through these pathways. In the pathways associated with proteins that were highly expressed in the preterm hAEC-EVs, the top three pathways are involved in the immune system, innate immune system and developmental biology which are associated with proteins 19, 13 and 12, respectively. This suggests that preterm hAEC-EVs may play a role in inflammation. What we found interesting is that Axon guidance protein was highly expressed in both term and preterm EVs, axon guidance proteins guide growing axons during development and control structural plasticity of synaptic connections. Changes in expression or function of these proteins induces pathological changes in neural circuits that predispose to, or cause, neurological diseases. This is an important finding in that EV therapy would be beneficial for preterm babies born with BPD who also develop cerebral palsy [67], as axonal injury is one of the hallmarks of cerebral palsy. Taken together, the significant differences in EVs protein cargo and the pathways that they are enriched in likely explain the functional differences between preterm and term hAEC-EVs.

We have previously shown that preterm hAECs had limited reparative effect in bleomycin-induced lung injury compared to term hAECs [68]. Hence, we did not assess the efficacy of preterm hAECs in treating BPD and, expectedly, we show that preterm hAEC-EVs did not have significant therapeutic effect in this BPD disease setting. We think that prematurity and complications associated with preterm birth may have influenced hAEC function and consequently the EV cargo and their functional potency. When bone marrow derived MSCs were preconditioned with 95% oxygen, the conditioned media contained higher levels of antioxidant stanniocalcin-1 and was more benefit for hyperoxia-induced lung injury rats compared to normal cultured MSC conditioned media [69], which implies that the changes of cargo in cell secretomes impact their functional potency. However, Bhandari et al. reported that the MSC EVs derived from umbilical cords of 25–30 weeks of gestational age exerted reparative effect in 95% hyperoxia-induced BPD mouse model by improving lung structure [70]. This improvement was attributed to TSG-6 (tumour necrosis factor-alpha-stimulated gene-6), because in their TSG-6 knockout mouse, MSC EVs had significantly reduced their therapeutic effects [70]. TSG-6 is an anti-inflammatory protein which plays an important role in immunosuppression of MSCs [71]. It has been shown to exert anti-inflammatory effects in an LPS induced lung injury model by shifting macrophage phenotype from M1 to M2 type [72]. Hence, we think future experiments investigating TSG-6 expression levels in hAEC-derived EVs would be of a great importance. Another study showed that the placental EVs derived from maternal peripheral plasma had declined bioactivity (endothelial cell migration area) in late pregnancy compared to early pregnancy [73]. However, this may not reflect the property of foetal stem/stem-like cells-derived EVs, as EVs derived from maternal peripheral plasma are a mixture of EVs from maternal and foetal origin, and factors such as secretion of EVs and placental perfusion need to be considered.

## Conclusions

Taken together, this is the first study to show that EVs isolated from term and preterm placenta have different cargo and functional potency despite sharing similar characterization for size distribution, morphology and exosomal markers. This study also demonstrates that term hAEC-EVs, but not preterm hAEC-EVs, have therapeutic efficacy in a mouse model of BPD-like lung injury. Term hAEC-EVs improved lung structure, reduced lung inflammation and had long-term benefit in improving lung function and preventing pulmonary hypertension and right ventricle hypertrophy.

There are increasing interests in EV application as a cell-free treatment. Compared to cell therapy, EV-based therapeutics have a number of advantages. As EVs are vesicles, the risks associated with administering live cells such as micro-vessel occlusion [74], infusion toxicities and ectopic tissue formation [75] are avoided. Furthermore, EVs may be stabilized for long-term storage [76], thereby enabling easier storage and low-cost cold storage solutions for international distribution [77]. The interest in the commercial development of therapeutic EVs has grown from about three companies (Caprocor Inc., ReNeuron Group PLC and Anosys Inc.) in 2014 to > 30 companies currently [78]. There are 20 registered clinical trials on the NIH website applying EVs as a therapy. It is worth noting that donor acceptance criteria and potency assays are very crucial to developing efficacious EV therapies. The International Society of Extracellular Vesicles (ISEV) released a paper regarding EV therapeutics in clinical trials in 2015, describing major considerations for the production of EV-based therapeutics. These include the characterization of the EV cell source, isolation and storage methods, quality control and in vivo analyses of EV potency [79]. The current study has unveiled the therapeutic differences between term and preterm hAEC-EVs in the setting of experimentally induced BPD, and this could be due to the different cargo they carry which may have different functional potencies even if they bear similar characteristics. We have previously shown that term hAEC-EVs in a bleomycin-induced lung injury model have therapeutic effects which were attributed to the protein cargo that was enriched in pathways for fibrosis specifically, PI3K-Akt, MAPK, Ras, Hippo, TGFb and focal adhesion signalling pathways [20]. We think differences in part between the therapeutic potential of preterm Vs term hAEC-EVs may be due to these factors. Further studies on the progenitor/stem/stem-like cells derived from gestational tissues of pregnancy complications are needed to uncover how pregnancy complications affect their therapeutic potential.

## Data Availability

All data generated or analysed during this study are included in this published article.

## Abbreviations

ALB: Albumin
α-SMA: Alpha-smooth muscle actin
ANOVA: Analysis of variance
APO: Apolipoproteins
BCA: Bicinchoninic acid
BM: Bone marrow
BASCs: Bronchioalveolar stem cell
BPD: Bronchopulmonary dysplasia
CTV: CellTrace Violet
cola1: Collagen alpha-1
E: Embryonic
EPCs: Endothelial progenitor cells
ELISA: Enzyme-linked immunosorbent assay
ECM: Extracellular matrix
EVs: Extracellular vesicles
FACS: Fluorescence-activated single-cell sorting
FiO2: Fraction of Inspired Oxygen
GM-CSF: Granulocyte–macrophage colony-stimulating factor
H&E: Haematoxylin and eosin
HSP: Heat shock proteins
HSCs/HPCs: Hematopoietic stem/progenitor cells
hAECs: Human amniotic epithelial cells
IL: Interleukin
ISEV: International Society of Extracellular Vesicles
LIF: Leukaemia inhibitory factor
LPS: Lipopolysaccharide
MIP: Macrophage inflammatory protein
MSCs: Mesenchymal stem/stromal cells
miRNA: MicroRNA
MISEV: Minimal Information for Studies of Extracellular Vesicles
MCP: Monocyte chemoattractant protein
NTA: Nanoparticle tracking analysis
NIH: National Institute of Health
PMA: Phorbol 12-myristate 13-acetate
PND: Postnatal day
PV loop: Pressure–volume loop
PAT: Pulmonary artery acceleration time
PET: Pulmonary artery ejection time
RANTES: Regulated upon activation, normal T cell expressed and presumably secreted
Crs: Respiratory system compliance
Rrs: Respiratory system resistance
RVAWT: Right ventricle anterior wall thickness
SEM: Standard error of mean
SDCBP: Syntenin binding protein
TF: Tissue factor
TEM: Transmission electron microscopy
TNF-α: Tumour necrosis factor-alpha
AT2s: Type II alveolar cells
UC-MSCs: Umbilical cord-derived mesenchymal stromal/stem cells
VEFG: Vascular endothelial growth factor
vWF: Von Willebrand factor
WJ: Wharton’s jelly
